# Secure UAV adhoc network with blockchain technology

**DOI:** 10.1371/journal.pone.0302513

**Published:** 2024-05-08

**Authors:** Mohammed A. Alqarni

**Affiliations:** Department of Software Engineering, College of Computer Science and Engineering, University of Jeddah, Jeddah, Saudi Arabia; University of the West of Scotland, UNITED KINGDOM

## Abstract

Recent advances in aerial robotics and wireless transceivers have generated an enormous interest in networks constituted by multiple compact unmanned aerial vehicles (UAVs). UAV adhoc networks, i.e., aerial networks with dynamic topology and no centralized control, are found suitable for a unique set of applications, yet their operation is vulnerable to cyberattacks. In many applications, such as IoT networks or emergency failover networks, UAVs augment and provide support to the sensor nodes or mobile nodes in the ground network in data acquisition and also improve the overall network performance. In this situation, ensuring the security of the adhoc UAV network and the integrity of data is paramount to accomplishing network mission objectives. In this paper, we propose a novel approach to secure UAV adhoc networks, referred to as the blockchain-assisted security framework (BCSF). We demonstrate that the proposed system provides security without sacrificing the performance of the network through blockchain technology adopted to the priority of the message to be communicated over the adhoc UAV network. Theoretical analysis for computing average latency is performed based on queuing theory models followed by an evaluation of the proposed BCSF approach through simulations that establish the superior performance of the proposed methodology in terms of transaction delay, data secrecy, data recovery, and energy efficiency.

## 1 Introduction

Recent technological advances in mobile aerial robotics and wireless communication and sensing technology have led to significant interest in micro unmanned aerial vehicles (UAVs), which integrate wireless sensors and transceivers in a compact node capable of autonomous flight operation and wireless communication. These UAVs find applications in various fields, such as logistics, cooperative communications [1, 2], mobile edge computing [3, 4], payload delivery [5], monitoring, surveillance, and search and rescue missions [6], to name a few [7, 8]. A single UAV is sufficient for simple tasks but more complicated missions require multiple UAVs to coordinate with each other as well as maintain continuous connection with ground nodes, such as IoT sensors/actuators or ground control stations, etc [9, 10]. These multiple UAVs form aerial networks that have no fixed backbone structure and are highly dynamic with vagaries of the often unreliable wireless channel making it very challenging to maintain formations and coordinate effectively [11, 12]. This is why these networks are called flying adhoc networks (FANETs) [13–15], an airborne variety of mobile adhoc networks (MANETs).

UAV adhoc networks are versatile and must be able to quickly respond and adapt themselves to evolving network environments [16, 17]. Maintaining a perfect mesh network is impossible in the face of fading wireless channels, interference, dynamic topology changes, and formations [18, 19]. It is easy to lose contact with the neighboring nodes and the network protocols must be resilient to these challenging conditions. Moreover, UAV adhoc networks are typically deployed in adverse situations, such as in post-disaster recovery zones, where the primary communication infrastructure is partially or completely dysfunctional [20]. UAV networks may also have to deal with potential threats from adversarial nodes, who might attempt to eavesdrop, intercept (man-in-the-middle attack), or compromise network coordination [21]. Ensuring the safety of the network nodes and preserving the privacy and integrity of data is paramount for accomplishing the objectives of the mission [22].

In this paper, we focus on the security of flying adhoc networks and propose a framework based on blockchain technology that provides security to the adhoc UAV network and ensures the integrity and privacy of the data exchanged among the nodes of the network. Blockchain technology provides a novel way to deal with privacy issues in exchanging data among several communicating parties [23, 24]. Blockchain is essentially a distributed database that allows reliable data storage using collaboration between nodes [25]. Blockchain technology could naturally be used to secure data exchange, via cryptographical transfers, for all users without even a trusted person through the features of decentralization, transparency, and data consistency [26]. It remains a major challenge to adapt blockchain methods for the variety of data handled by the UAV networks in terms of security and latency constraints [27, 28]. In the proposed method, we differentiate data generated by the network nodes in terms of their urgency and security requirements. The blockchain is then customized to tradeoff among these requirements to facilitate faster transaction processing, high average secrecy levels, high data recovery rate, and greater energy efficiency.

The integration of various technologies, such as IoT, machine learning, blockchain, cloud computing, and smart communication technologies makes the overall system efficient and secure [29].

The primary contributions of the proposed framework in this work are as follows:

- A blockchain-assisted secure framework (BCSF) is proposed to protect the privacy of data exchanged over an ad-hoc UAV network. The proposed system leverages cryptographic hashing and other features of blockchain to ensure the security and privacy of data.
- The proposed framework is optimized for data representing different levels of urgency and requiring different levels of security. The latency of the overall network is determined theoretically for an M/M/1 queuing model.
- The performance of BCSF is evaluated by simulating the UAV ad-hoc network and gradually scaling the network size. The proposed framework exhibits efficiency in terms of transaction time, recovery rate, secrecy rate, and energy efficiency.

The remaining article is organized as follows: Section 2 comprises various background studies concerning the use of blockchain technology in ad-hoc UAV and IoT networks. Section 3 proposes the BCSF model for ad-hoc UAV model and presents a theoretical analysis of the proposed approach using M/M/1 queuing model. Section 4 constitutes the results that validate the performance of the proposed model with the corresponding descriptions. Finally, the conclusions are drawn in section 5.

## 2 Related work

This section discusses several relevant works in which researchers have proposed the use of blockchain technology to provide security to network data.

Tang et al. [30] propose a secure mobile-edge computing system that ensures the privacy of user data during the computation offloading process. The authors combine a block coordinate descent algorithm with a successive convex approximation method to generate optimal trajectories for UAVs. The proposed secure computing architecture combines mobile-edge computing with blockchain to provide privacy and security to user data during computation offloading between mobile and UAV users.

Jian Wang et al. [31] introduced blockchain-assisted secure routing (BASR). BASR is a compact, stable routing scheme based on blockchain for unmanned Aircraft System (UAS) communication swarm. BASR uses a lightweight blockchain to improve swarm UAS communication safety based on 5G NR technology. The assessment shows that Proof-of-Traffic (PoT) decreases connectivity utilization in consensus building and block coordination phases. The lightweight Blockchain-assisted approach proposed in this paper, consistent with the restricted swarm tool of UASs, can retain swarm UAS communication performance.

Abdur Rahman et al. [32] discussed the blockchain-managed federated learning approach (BMFLA). BMFLA introduces a compact hybrid architecture in which a decentralized smart grid controls the edge training schedule, trust maintenance, and encryption of interacting federated nodes. The frame facilitates the complete encryption, model construction, and referencing mechanism of a dataset. The model also has support for the training and inferencing process.

Rupa et al. [33] present a blockchain-based solution to improve the security and privacy of device data in a UAV or drone-assisted IoT network. The proposed design is evaluated for a vehicle monitoring system and uses Elliptic curve cryptography (ECC) and SHA to provide privacy guarantees. Data is stored in Ethereum based public blockchain to enable blockchain transactions. The proposed method is efficient and secure and protects device data against plaintext and ciphertext attacks.

J. Indumathi et al. [34] proposed the use of blockchain for a network composed of IoT devices. The proposed approach takes advantage of blockchain’s benefits such as reduced expense, transparency, flexibility, almost unnecessary data loss, longevity, elimination of mediators, centralized control, data protection, and the effectiveness of supply chain management. The layered Architecture results are checked and proved competent to achieve secure audits and exceed those of the previous ones.

Anik et al. [35], propose the use of UAVs to assist an IoT network in the acquisition of data. The UAVs help with better coverage, efficient data transmission, energy efficiency, and provide security and privacy through the use of blockchain technology. Mobile edge computing server in the cloud receives encrypted data from IoT devices with UAVs acting as relays. Blockchain helps with the integrity of data and pseudonymity. MEC server can use a blockchain consensus algorithm to ascertain the identity of the sender and validate the data.

Gupta et al. [36] propose a blockchain-assisted secure system for UAV communication in a 6G environment. The authors emphasize that the UAV network has vulnerabilities and its susceptible to cyberattacks from adversaries. Authors proposed a blockchain-based method that has low latency and bandwidth and can operate over 6G cellular networks. The proposed approach guarantees security and privacy of data transmitted over the UAV network.

Based on the survey of related work, a block-chain assisted security framework (BCSF) has been proposed to safeguard data exchange among the nodes in an ad-hoc UAV network. The proposed framework with privacy protection is built on blockchain technology. In the next section, we describe the proposed framework followed by the evaluation of the proposed framework, i.e., BCSF in terms of energy efficiency and delay incurred in generating a transaction.

## 3 Proposed methodology

The introduction of blockchain to an adhoc UAV network significantly improves the security, transparency, and availability of data under adverse circumstances and threats to the network structure that would be determinantal in a centralized hierarchical network. For faster, secure, and dependable delivery of data, it is important to exploit the decentralized and distributed nature of the database offered by blockchain technology. UAV networks are typically deployed in an emergency or to carry out critical missions. These activities are typically carried out under adverse circumstances in areas that are hostile to other possible actors who possess conflicting objectives and intend to harm the UAV adhoc network and compromise the integrity and security of data. Therefore, it is vital to adopt security countermeasures to thwart such threats by removing vulnerabilities within the network. Many UAV operations involve saving human lives and handling sensitive data transmission and exchange among the network entities, such as other UAVs and the ground stations. So it is critical to ensure the privacy and security of data and infrastructure within the network. Moreover, UAVs networks face constraints that are usually not present in other networks, e.g., due to size and weight limits, they have limited battery life, which is required for both mobility and data transmission and reception. Due to these constraints, the efficiency in processing data onboard and communicating data among the UAV devices and the ground station is highly desirable. In this paper, we propose a blockchain-based security framework (BCSF) paper that aims to safeguard data acquired and exchanged by the nodes of a UAV adhoc network while ensuring the efficiency of processing and transmission within the overall network. The proposed scheme provides privacy, security, and recovery of data through the distributed nature of blockchain technology. The proposed scheme first outlines the circumstances and constraints of the data processing operations in the adhoc UAV network and subsequently specifies network design and policies relevant to these constraints. In this section, we develop a tailored transaction structure that fits UAV data with storage-limited blocks to maintain data secure and confidential. We demonstrate the efficiency of the proposed framework by considering a sample data vector to be exchanged among the nodes on the network.

**Fig 1:**
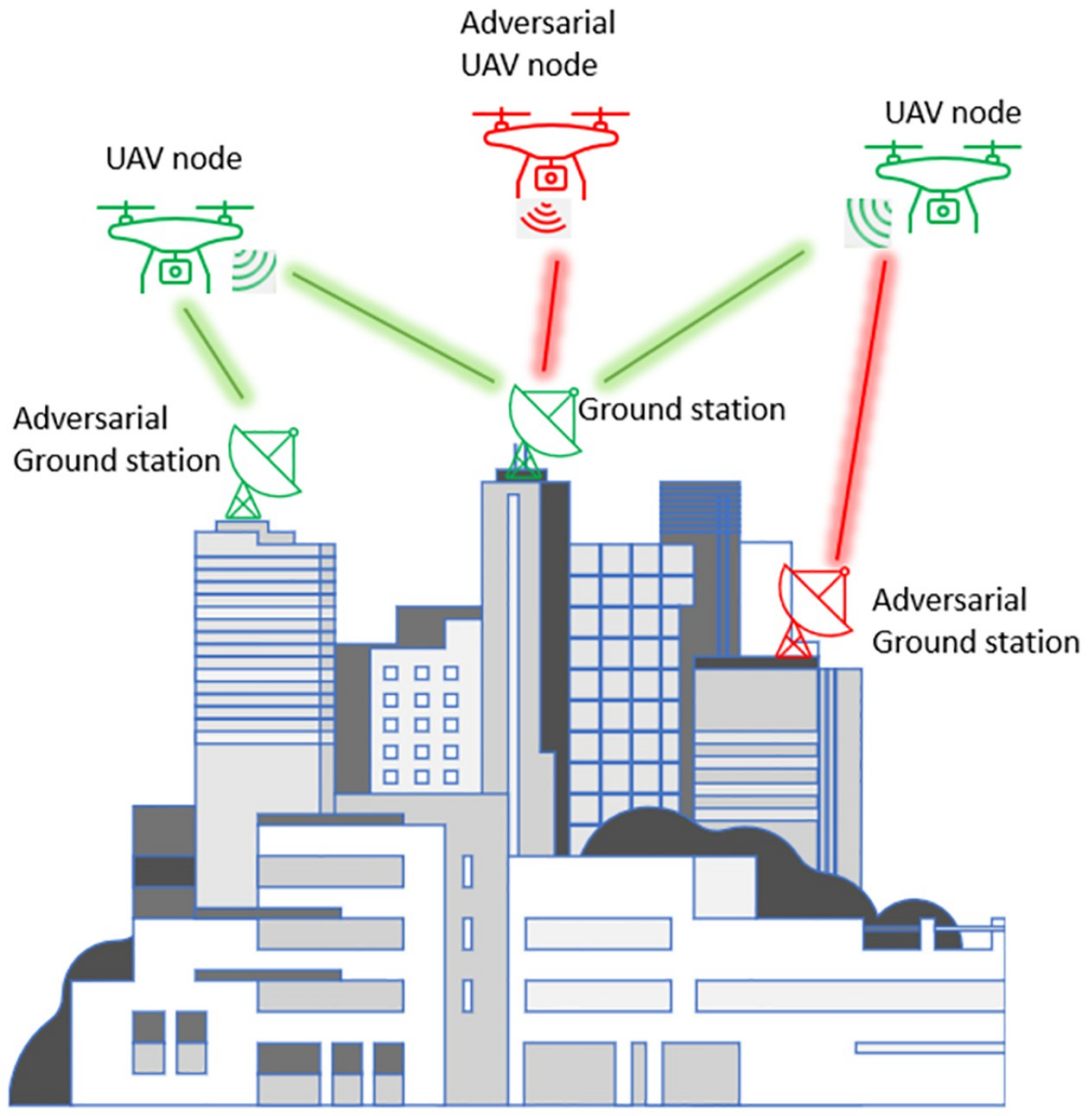
An example adhoc UAV network facing adversarial actors attempting to launch a man-in-the-middle attack.

Fig 1 shows an example adhoc UAV network, also referred to as a flying adhoc network (FANET). UAV adhoc networks may provide data collection services from a variety of internet-connected devices deployed aerially or on the ground or the UAV nodes themselves might act as an information source or sink within the network. The relationship between UAV devices and sensors may allow the network operators to improve the coverage and availability of their services or to perform a mission considered impossible or too dangerous without mobile robots. UAV networks are typically deployed in hostile or remote areas where there is either no primary network available or the network is unavailable due to a disaster or emergency. Furthermore, the use of a public network might be undesirable as it may increase the threat to the security of an adhoc network or may introduce latency that might be intolerable and violate the response-time constraints of the mission. The objective of the UAV network is to provide emergency services, such as an initial estimate of the scope of the search and rescue mission. This requires the network to adapt in real-time to changing network structure, e.g., with nodes leaving and entering the network, it is critical to authenticate the source of data. Blockchain consensus algorithm ensures that the data is immutable and its integrity is guaranteed against limited attacks. The network of drones connects the UAV nodes among themselves as well as to the ground stations that might be connected to the cloud for data processing, analysis, and decision-making in real-time. The safety and security of the network against threats is paramount to the success of the mission. UAV nodes also exchange flight data and its geo-location with each other to synchronize activities among various UAV nodes in missions that involve collaboration among multiple agents. The integrity and confidentiality of this data and its immutability is equivalent to the successful attainment of the mission objectives. By exchanging information over a secure network, UAV devices interconnect stakeholders, operators, data sensors, actuators, and infrastructure elements within the environment. Real-time efficient processing and exchange of data without compromising its security extends the lifetime of the network and puts less stress on the limited resource nodes within the UAV network.

**Fig 2:**
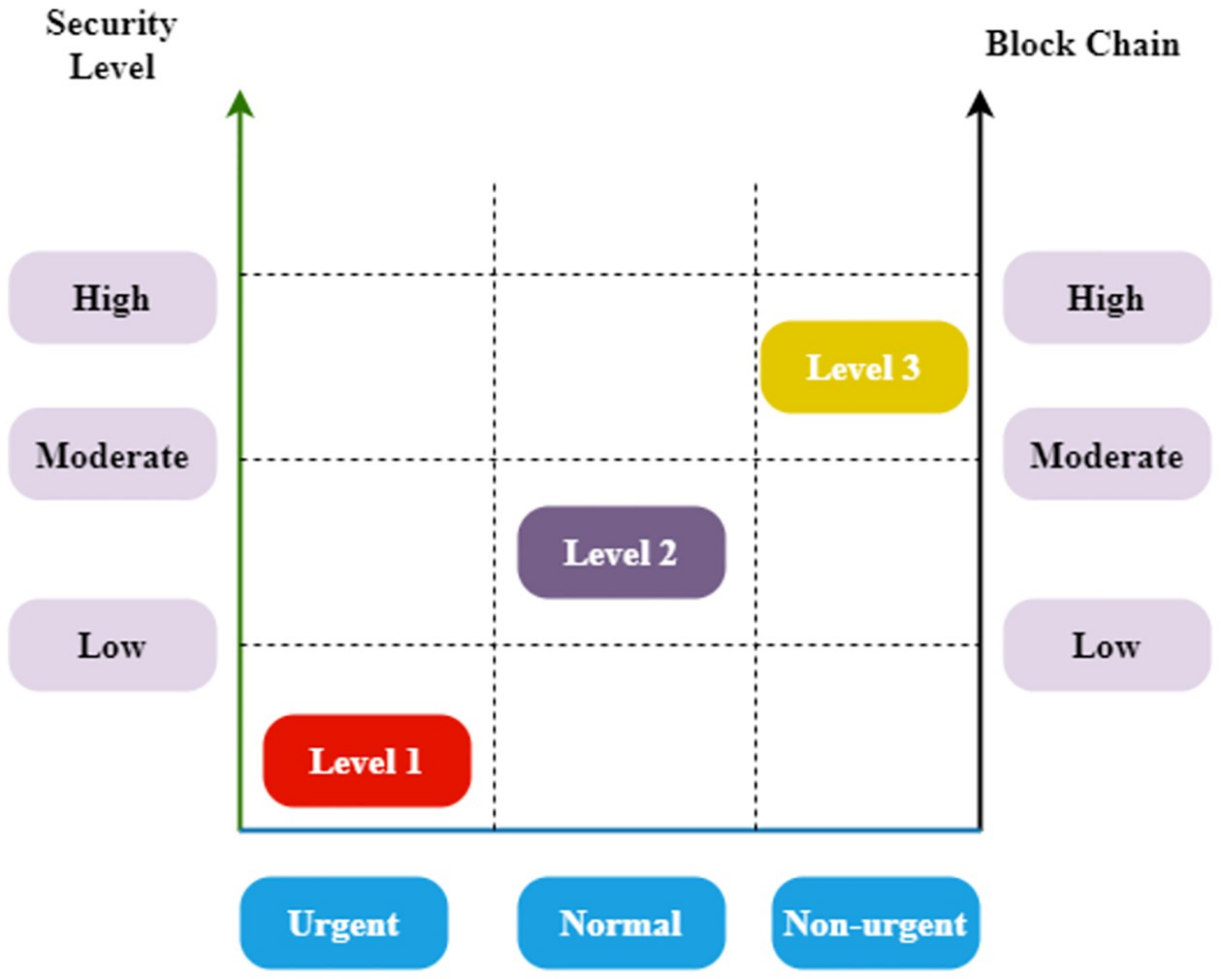
Security level encryption.

Fig 2 explores the security level encryption. The second phase of our framework is implementing an optimized blockchain model that allows different mission-related events and information to be shared between different entities on the network. The proposed method assumes that data obtained from different entities might be handled differently to design an efficient network based on their emergency and safety levels. For example, the most priority should be given to urgent data, and a less limited blockchain, i.e., a minimum of validators for consensus, is selected. Conversely, a completely restricted blockchain can be used for low-priority data types with a high-security level. For regular data, i.e., with latency and security requirements, an optimized blockchain setup is used. Note that various data classification, event detection, and summary strategies apply to defining data types and the emergency levels at the edge.

Consider the importance of the blockchain manager (BM) priority assignment to maximize blockchain configuration parameters. This task aims to reduce the delay in completing the transactions in accordance with their time-related constraints and urgency. In this connection, the stay time refers to the transaction’s total time to wait before a block is augmented to the blockchain. Different levels of emergency, namely urgencies, regular and non-urgent, can monitor this stay time. Then use queuing models to determine the residence time based on the urgencies of various transactions obtained. To determine the residence period using the priority concept for preventive resume transactions, transactions of greater priority disrupt the processing of lower priority transactions.

Let us assume that *M* entities (e.g. a UAV or a sensor within the network) send their transactions to a BM, and let *j* ∈ {1, …, *M*} be the index over these transactions with the respective arrival rates referred to by *μ*_*j*_. All transactions obtained from various entities are stored in a BM buffer temporarily. Now $\sum_{j=1}^{M}\mu_{j}$ represents the rate of services at the BM (we assume that the buffer memory is large enough to prevent any buffer overflows from happening).

When the developed *M*/*M*/1 queuing model for the transactions obtained with equal priorities is adopted, the average stay time of the entity *j* is defined as

$$\begin{array}{c} T_{j}^{e}=\frac{1}{\lambda -\sum_{j=1}^{M}\mu_{j}}. \end{array}$$ (1)

As shown in (1), the average time is different from the time experienced by individual entities. BM sets different priorities for different entities based on their urgency levels and the corresponding weight for an entity is used to manage the transactions received from it effectively. Therefore, the highest priority would be attached to transactions of high urgency from high-impact entities. Let’s denote the residence time by $T_{j}^{h}$, which can be computed by applying the average stay period for transactions with different priorities in (2):

$$\begin{array}{c} T_{j}^{h}=\frac{\sum_{j=1}^{M}\mu_{j}O_{j}}{\left(1-(\frac{\mu_{1}}{\lambda }+\ldots +\frac{\mu_{j}}{\lambda }))(1-(\frac{\mu_{1}}{\lambda }+\ldots +\frac{\mu_{j-1}}{\lambda })\right)}+\frac{A_{j}}{1-(\frac{\mu_{1}}{\lambda }+\ldots +\frac{\mu_{j-1}}{\lambda })}. \end{array}$$ (2)

As shown in (2), the average stay time has been obtained, where the mean service is *O*_*j*_ or *A*_*j*_ and the mean *j*^th^ entity residual service hours. The implemented *M*/*M*/1 queuing model means that for exponential service times, $A_{j}=\frac{1}{\lambda }$ and $O_{j}=\frac{1}{\lambda }$. The above results replace the yields of the following average stay time expression in (3):

$$\begin{array}{c} T_{j}=\frac{\frac{1}{\lambda }\sum_{j=1}^{M}\mu_{j}}{\left(1-(\frac{\mu_{1}}{\lambda }+\ldots +\frac{\mu_{j}}{\lambda }))(1-(\frac{\mu_{1}}{\lambda }+\ldots +\frac{\mu_{j-1}}{\lambda })\right)}+\frac{\frac{1}{\lambda }}{1-(\frac{\mu_{1}}{\lambda }+\ldots +\frac{\mu_{j-1}}{\lambda })}. \end{array}$$ (3)

As obtained in Eq (3), the average stay time expression has been found. To evaluate the advantages of the suggested assignment of an urgent priority compared to traditional approaches that use equal priority assignment, $\lambda =\frac{m}{K}$, where *m* is the number of a block of the transaction, and *k* is the block latency inside the blockchain. Thus, the optimization of blockchain settings would directly affect the stay obtained.

The consensus algorithm adopted in the proposed method is the delegated proof of stake (DPoS) [37], which uses immediate post-validators to carry out the consent process. The three primary metrics in our model at BM are latency (*K*), safety (*ζ*), and costs (*D*). These metrics thus have several distinct values and units to be normalized about their maximum levels (referred to respectively by *k*_*n*_, *ζ*_*n*_, and *d*_*n*_) to make them comparable. Next, we define an aggregate utility *V* to handle such conflicting metrics and combine them into a single feature:

$$\begin{array}{c} V=\sigma \frac{K}{k_{n}}+\beta \frac{\zeta_{n}}{\zeta }+\alpha \frac{D}{d_{n}}. \end{array}$$ (4)

As found in (4), utility of the function has been expressed in terms of relative significance *σ*, *β*, and *α* of the metrics considered, such that *σ* + *β* + *α* = 1. Furthermore, *n* is the number of selected validators, with minimum values and maximum equal to *τ* and *χ*, respectively, and *m* is the number of transactions per block, with minimum and maximum values equal to *S* and *Y*, respectively. The best blockchain configuration is, therefore, possible for the BM by resolving the following optimization problem in (5):

$$\begin{array}{c} min_{m,n}V\\ D_{j}\geq \epsilon_{j}Y_{j},\\ \tau \leq n\leq \chi ,\\ S\leq m\leq Y. \end{array}$$ (5)

The optimization problem defined in (5) uses *D*_*j*_ as the computational cost, *ϵ*_*j*_ is the validator cost to complete the verification work *m*, with security level defined as $\zeta =\theta_{n}^{p}$, where *θ* is the system coefficient and *p* ≥ 2 is a network scale indicator factor. *K* corresponds to the delay of the block validation process, including

- the unauthorized BM block transmissions of validators;
- the block validations time;
- the outcome verification of diffusion and contrast relationship of validators; and
- the input transfer from the validators to BM.

The latency is thus defined in (6):

$$\begin{array}{c} M=\frac{m.A}{O_{c}}+max_{j\in \{n,\ldots ,m\}}(\frac{L}{Y_{j}})+\psi \left(m.A\right)n+\frac{R}{O_{v}}, \end{array}$$ (6)

where *A* is the transaction size, *L* is the computational resource needed for a block verification task, *Y*_*j*_ is the computer resources available at the validator *j*, *R* is input size, *O*_*c*_ and *O*_*V*_ are the downlinks and uplinks of BM transmission rates to the validators, and vice versa. *ϕ* is a pre-defined parameter to be obtained using statistics from previous block verification processes. Finally, in our architecture, validators *j* are believed to download their computer load *Y*_*j*_ for cloud/fog providers (CFPs) from the verification process. These *Y*_*j*_ from CFP should obtain the costs *D*_*j*_ at least cover payments to the CFP to access these resources from the distant cloud and the nearby fog computing unit’s nearby validators to engage in the verification process. This is a constraint in which *ϵ*_*j*_ is the validator *j* payment to the CFP to obtain the necessary resources for the verification process.

**Fig 3:**
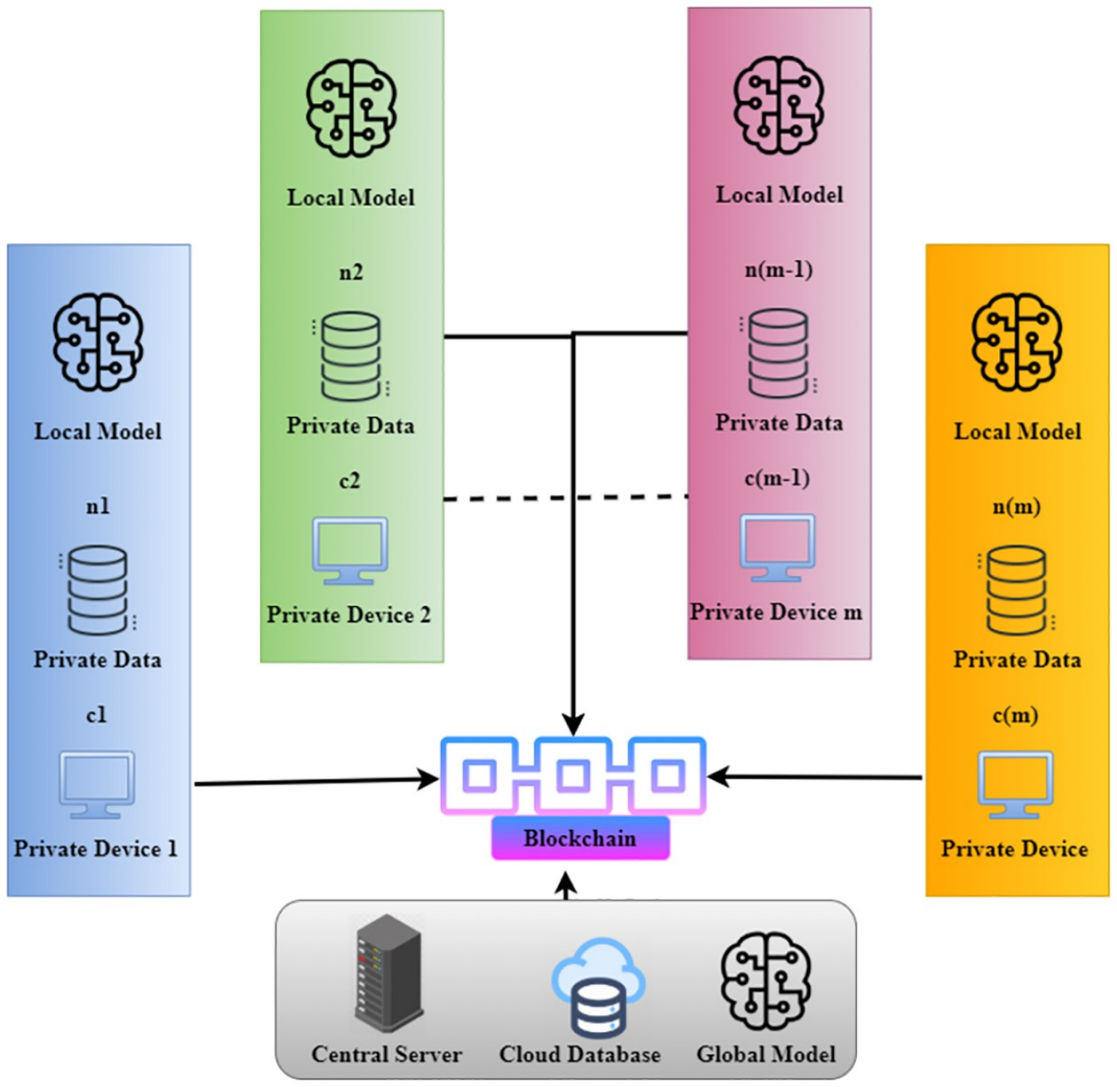
Blockchain-based private device data.

Fig 3 demonstrates the blockchain-based private device data. The architecture is built within the BCSF and is split into multiple sections, including the global model, local model, blockchain, cloud, and central server. The security framework is considered an iterative process in which the fundamental model is upgraded in each iteration. The first step is to share the worldwide model with the stakeholders. Secondly, the initial device model (also known as a local model) will be improved with personal data training, eliminating the need for real data to be sent to the server. Finally, device-level training is provided for the local models, and the transaction forms stock updates (model parameters). The central server reads the parameters from the blockchain to aggregate and train the global system. The global model is revised, and for the next iteration, the enhanced model is shared between the individual devices. The blockchain network will consist of this architecture of global participating nodes involved in transaction verification. There are field-specific, technology-dependent choices for the participating node and the consensus mechanism to be addressed. All samples are taken to the AI component, where the following identified samples are added to the database at *n*_1_, *n*_2_, …, *n*_*m*_, and all data is used in training. In the second architecture, the balanced setting would be overwritten if post-training better performance is obtained. With two theories of this same classification, the basic architecture with weights and classification parameters is stored in a local computer, where the global model is downloaded. Databases and user data protection via blockchain allow *d*(*n*) access to device-specific events. Additional encryption or key security will provide their safety. Firstly, the operation of methods should be improved at individual data analytics stages. It’s worth noting that the data-hungry algorithm is called the artificial intelligence method, and with a large amount of data, it is much more efficient and precise.

Hashed information is added to the range to have a length of 1024 bits. The following step divides the message into 1024-bit blocks and marks the message as *N*^(1)^, *N*^(2)^, …, *N*^(*M*)^. When blocks have been constructed this way, presume that the starting hash quantity is known and labeled as

$$\begin{array}{c} G^{\left(M\right)}=G^{\left(j-1\right)}+D_{N^{\left(j-1\right)}}\left(G^{\left(j-1\right)}\right), \end{array}$$ (7)

where *D* is expressed as a compression feature. A convolutional neural network is a type of classification inspired by the concept of data processing. There are three kinds of layers of the mathematical model. The first is referred to as convolutional, which is interpreted as a filter application on the data. This layer type intends to find data features and is considered a map after this layer. The next layer consists of a single layer, which reduces the data size by maximum function. The highest value is moved to the next layer for each sample data vector. Classic neural network architecture is completely linked with the last layer type. The vector has been flattened and transferred to a completely connected pixel of the last pooling layer. Neurons that calculate the active impulses and forward them are defined in this layer as follows:

$$\begin{array}{c} E(\sum_{i=0}^{L}\sum_{j=0}^{L}Z_{j,i}.B_{y+j,x+i}). \end{array}$$ (8)

The neuron activation function is initialized as mentioned in the equation above, Where *E* is the activation function of a neuron *B*_*y*,*x*_ and where (*x*, *y*) implies its position, *L* is the filter matrix. Each of the synapses has weights *Z*_*j*,*i*_. In the training process, these values are modified to determine the best settings for defined issues. In each iteration, the weight will be changed, and the loss function will be reduced. Statistical values, such as mean *n* and variance *v* with *γ*_1_ and *γ*_2_ are defined as in this algorithm in Eq (9).

$$\begin{array}{c} n_{s}=\gamma_{1}n_{\left(s-1\right)}+\left(1-\gamma_{1}\right)h_{s}^{1},\\ v_{s}=\gamma_{2}v_{\left(s-1\right)}+\left(1-\gamma_{2}\right)h_{s}^{2}. \end{array}$$ (9)

These statistical values are used to calculate the correlation after these two values have been normalized:

$$\begin{array}{c} {\hat{n}}_{s}=\frac{n_{s}}{1-\gamma_{1}^{s}},{\hat{v}}_{s}=\frac{v_{s}}{1-\gamma_{2}^{s}}. \end{array}$$ (10)

These correlation factor values are used in neural network weight-changing formula as follows:

$$\begin{array}{c} Z_{s+1}=Z_{s}-\frac{\zeta }{\sqrt{{\hat{v}}_{s}}+\epsilon }{\hat{n}}_{s}, \end{array}$$ (11)

where *ζ* represents the rate of learning, and *ϵ* is a small value that prevents division by zero. The device-specific data is used to predict future values and perform further analysis, depending on the data’s form, using artificial intelligence techniques. Everything is being re-analyzed. These numbers are from the range [0, 1] for each input data vector returned for each classification. For every two data values on different occasions, the results are compared as follows in the equation below:

$$\begin{array}{c} L=j_{1}-j_{2}=[\begin{array}{c} O_{1}^{j_{1}}\\ O_{2}^{j_{1}}\\ \vdots \\ O_{m}^{j_{1}} \end{array}]-[\begin{array}{c} O_{1}^{j_{2}}\\ O_{2}^{j_{2}}\\ \vdots \\ O_{m}^{j_{2}} \end{array}]=[\begin{array}{c} O_{1}^{j_{1}}-O_{1}^{j_{2}}\\ O_{2}^{j_{1}}-O_{2}^{j_{2}}\\ \vdots \\ O_{m}^{j_{1}}-O_{m}^{j_{2}} \end{array}]\;, \end{array}$$ (12)

where *j*_1_ and *j*_2_ mean two separate data results (*j*_1_ before *j*_2_) and *O* values for individual classes are interpreted as the output value. The findings are then analyzed through the detection of the biggest abnormalities. The result is a set of {*L*^1^, *L*^2^, …, *L*^*n*^} data vectors. The maximum value is found in each line, which allows the following vector to be constructed:

$$\begin{array}{c} {}[\begin{array}{c} max(L_{11}^{1},L_{11}^{2},\ldots ,L_{11}^{n})\\ max(L_{21}^{1},L_{21}^{2},\ldots ,L_{21}^{n})\\ \vdots \\ max(L_{m1}^{1},L_{m1}^{2},\ldots ,L_{m1}^{n}) \end{array}]\;. \end{array}$$ (13)

If the values in the vector above are large, they signify time changes that can be interpreted as gradual action in a certain class. For numerical values, the same operation can be performed. The analysis will be undertaken collectively in both classes and the structure of the classification classes.

The sample data vector could represent different things in a drone adhoc network such as sensor measurements and also possibly an image. First, we define the usual data retrieval situations and then summarise the corresponding specifications for a system to protect the confidentiality of the data vector. Multi-data sources intelligent data retrieval: The system should allow data recovery from a collection of data samples from various sources. The proposed method focuses on exchanging sensor and UAV data. Cloud can be used to collect information from the platform, and machine learning-based analysis algorithms will upgrade the accuracy of prediction models by constantly increasing data resources.

Now we discuss system model and threat model, of which the first includes the layered architecture of the proposed scheme. The structure is customized to acquire an appropriate data vector in the transaction layer. The data statistics are computed in the service layer, and smart contracts carry out the recovery process. In the future, appropriate scenarios will be extended in the application layer to include data modeling. The sensors and UAVs could be the source of the data vector. First of all, sensor modules or UAV devices extract the features of data vector. These features will then be encoded and blockcast using Secure Multi-Party Computing with the corresponding diagnostic information. If a third party requests data, relevant data characteristics should be removed to make it anonymous. The smart contract loads the query demand for processing the data recovery service. The third-party finally obtains the results of the search, including similar encoded data. The data retrieval service encrypts and stores a data vector on the blockchain using retrieving and downloading the public addressed to confined databases. Every data is an index to improve recovery productivity. The data recovery service dynamic replaces the index. There is a smart contract code deployed over it. If the user provides a data recovery query, the code automatically performs the required operations, and the data encoding function closest to the data is discovered. The regulatory body (RA) is responsible for checking the legal aspects of the transaction and third-party authentication information. When entering this platform, users of the system must register with the RA and enter their authentication information. It is the first guard to guarantee the safety of a system. This prevents unwanted nodes from manipulating transactions and or from launching attacks on smart contracts. The miner monitors the completeness of blockchain data. The encrypted data information downloaded from the database contains a digital hash signature, a 128-bit string formed from SHA256’s encrypted data. With its private key, the authorized user can create a digital signature. Afterward, the digital signature loads encrypted data into the blockchain and provides a public key to the miner. The miners use the network administrator’s public key and hash algorithm to verify its encoded hash signature. If the encryption function is uploaded to the blockchain, any potential threats posed to the facility will be received, or users will request access. They will hurt the privacy of data directly from the blockchain. The smart contract accurately encodes the data and provides the user with the encrypted results.

The corresponding ciphertext *D*_*j*_ can be computed for each element of the original vector *E* using the equation below:

$$\begin{array}{c} D_{j}=\left(1+b_{j}Q\right)O_{j}modQ^{2}. \end{array}$$ (14)

After a round of exchanges of information, *Q*_*j*_ can eventually compute $\hat{E}$ of the vector *E* function. Until all ciphertext data has been collected, each UAV will spread ciphertext to one another. Finally, it is possible to compute the total ciphertext. The proposed system BCSF has been designed to improve the adhoc UAV network’s performance and enhance user accessibility to achieve improved transaction generation time, security rate, data recovery rate, better cost function, enhanced packet transmission rate, low latency, and energy efficiency.

## 4 Results & discussion

**Fig 4:**
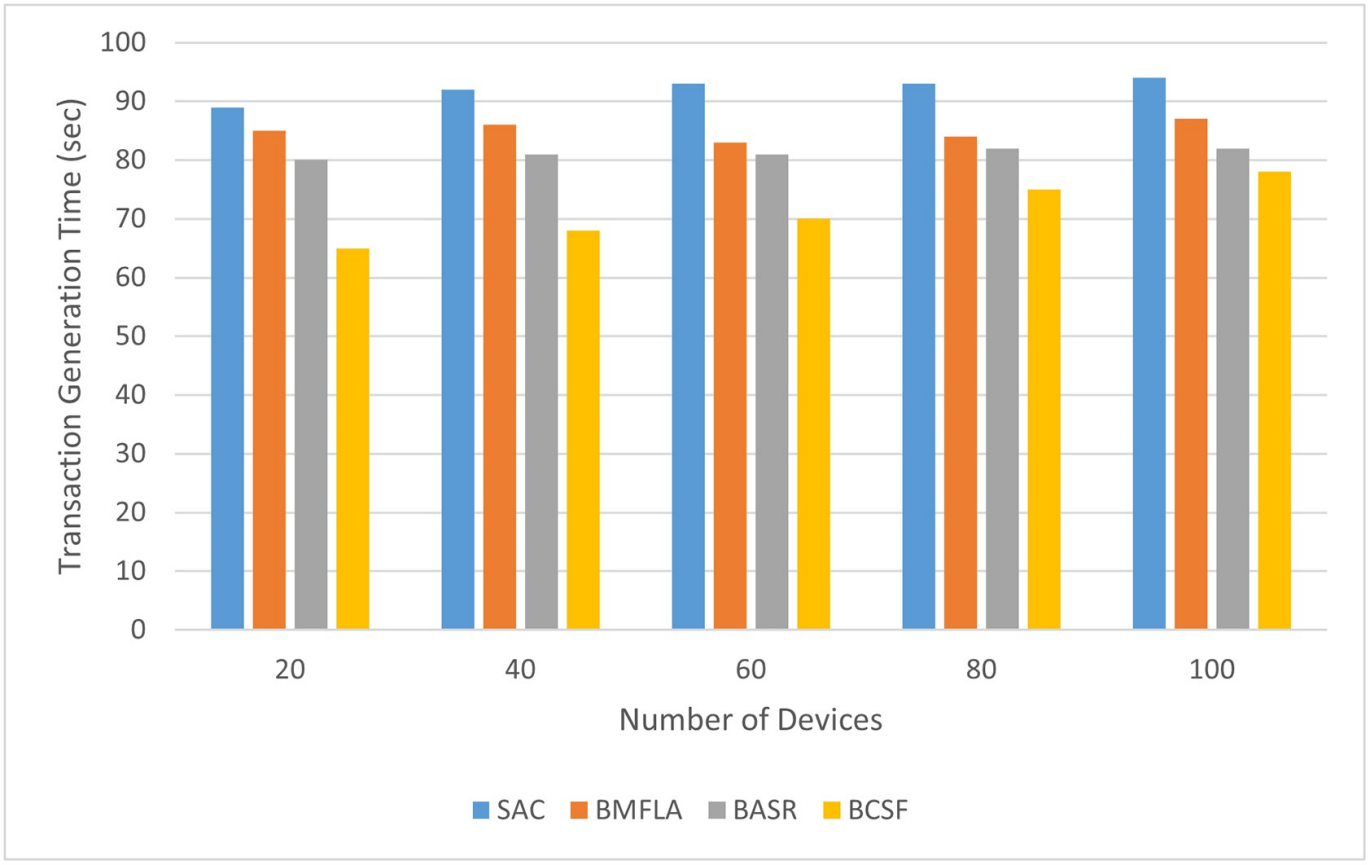
The transaction generation time of BCSF comparison to other techniques with varying number of devices.

The proposed BCSF has been validated based on data security level and retrieval time. The time required for transactions with differing capabilities (represents the number of data to be transferred in one transaction). Generally speaking, the time to create the transaction should be less than a few cycles, ensuring the user can effectively exchange their details on the blockchain system. The transaction generation time of BCSF is shown in Fig 4. It can be observed that the transaction generation time increases in general with the increasing complexity of the network. However, the transaction generation time for the proposed algorithm is short as compared to SAC, BMFLA and BASR techniques.

**Fig 5:**
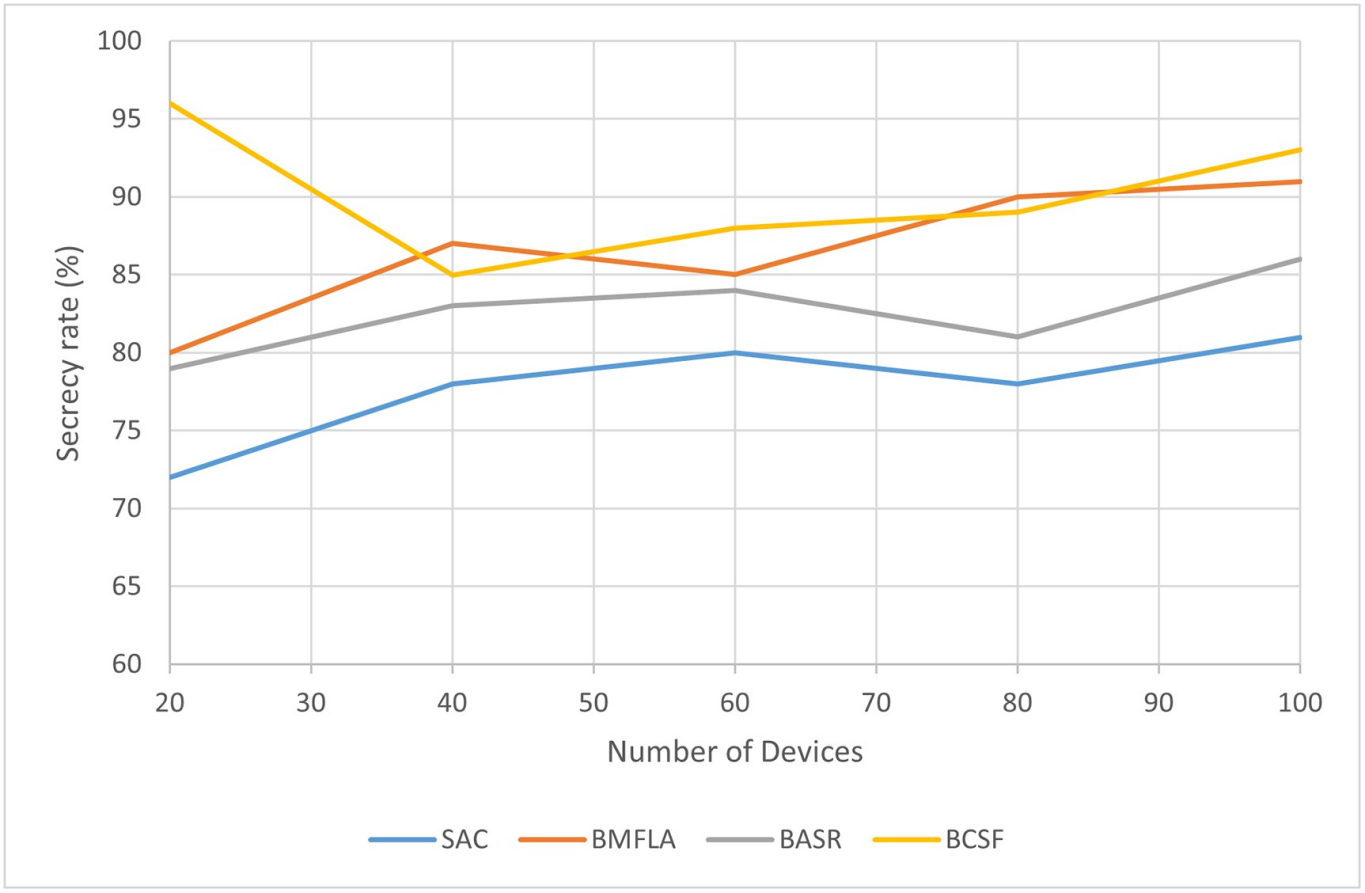
The data secrecy rate of BCSF compared to other techniques with varying number of devices.

Data authentication is an effective way to avoid data protection disclosure. There are initial documents with an encryption key for each node. Although not each node wishes to receive content details from unauthorized access, the secret key is not being released. The Blockchain is usually used during data manipulation. All users must report and submit their identification details to maintain the data suppliers’ security and reliability. The data security of BCSF is shown in Fig 5. The figure shows that the proposed BCSF algorithm has superior performance when the number of devices are less. However, as the number of devices increases, the performance of BFMLA algorithm also improves and it approaches the BCSF algorithm.

**Fig 6:**
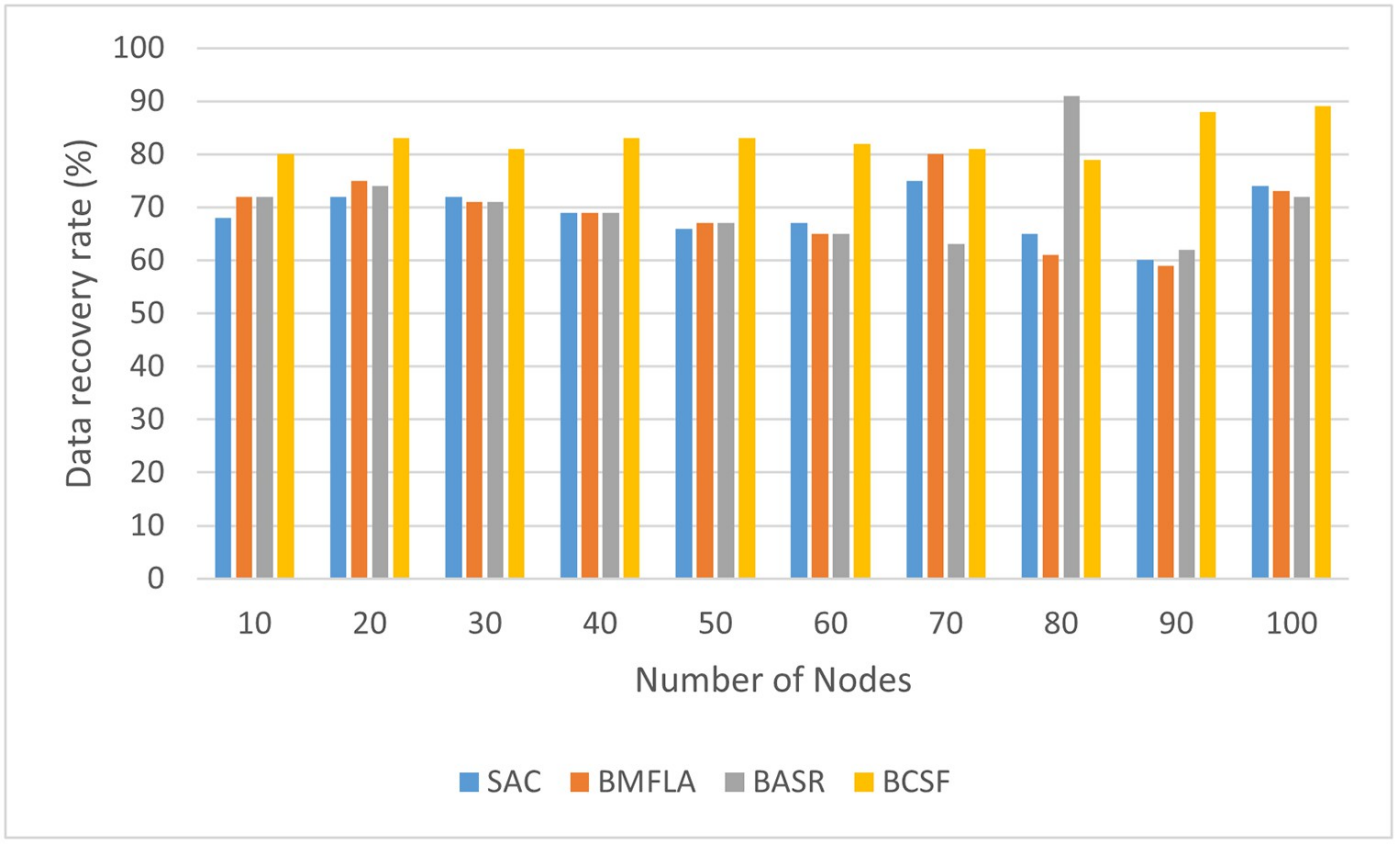
The data recovery rate of BCSF compared to other techniques with varying number of devices.

The data recovery rate is shown in Fig 6. The period from submitting a data retrieval query is the user’s time to receive the results obtained. The retrieval application is processed in a few phases, including queries sent, verifying the query through an intelligent contract, and measuring data similarities and response results. The duration required to retrieve the possible framework at an average recovery time is fairly constant. It must be mentioned that performance from a data size and service bandwidth standpoint could be further improved. Fig 6 shows that the performance of the BCSF algorithm and BASR algorithm leads as the number of nodes increases as compared to the BMFLA and SAC algorithm.

Another important output factor for tracking the enhancement of the mean energy consumption is effective energy efficiency. For encryption and transfer of data, the collection of unique mining has been used. The overall energy efficiency of the BCSF under the proposed approach, as shown in Table 1. is more than the current method. This can be attributed to the fact that the existing algorithms check any block with a particular generator with the minimum amount of energy.

**Table 1:** Comparison of effective energy efficiency (%) for data transmission.

Number of Devices	SAC	BMFLA	BASR	BCSF
20	79.1	72.3	70.1	80.2
40	75.2	83.7	70.2	84.3
60	80.5	71.3	70.9	81.3
80	87.6	69.2	78.2	87.4
100	85.1	66.2	74.1	87.1

The proposed method achieves the highest data secrecy rate, good data recovery rate, fast transaction generation time and high energy efficiency when compared to existing methods, such as secure aerial computing (SAC), blockchain-managed federated learning approach (BMFLA), and blockchain-assisted Secure Routing (BASR).

## 5 Conclusion

This paper presents blockchain-assisted security framework (BCSF) to safeguard the privacy of data communicated over the adhoc UAV network. An energy-efficient data recovery system with privacy protection is proposed based on blockchain technology. Faster data generation with high energy efficiency is very important for the secure operation of the UAV-based network due to its power constraints and mission-critical objectives. UAV networks pose significant operational challenges due to their high mobility and dynamic structure that evolves rapidly in space and time according to the assigned objectives. Synchronization and coordination of UAV adhoc networks require fast and efficient mechanisms for data exchange, transmission, and recovery so that flight control and formation control can be achieved while meeting the latency and energy constraints on the network nodes. The proposed method is based on blockchain technology and its average transaction time is optimized based upon the priority parameters assigned to a sample data vector. The adhoc UAV network also benefits from the immutability, authentication, and security of the blockchain implementation. For information exchange over this network, we propose a customized transaction framework for large-scale data with blocks that keep data secure. The experimental result suggests that BCSF provides a high average data protection level, fast transaction generation time, high rate of secrecy, and very good energy efficiency and recovery time when compared to other methods.

## Supporting information

S1 Data (XLSX)
